# Antidepressant use and cognitive decline in community-dwelling elderly people – The Three-City Cohort

**DOI:** 10.1186/s12916-017-0847-z

**Published:** 2017-04-19

**Authors:** Isabelle Carrière, Joanna Norton, Amandine Farré, Marilyn Wyart, Christophe Tzourio, Pernelle Noize, Karine Pérès, Annie Fourrier-Réglat, Karen Ritchie, Marie Laure Ancelin

**Affiliations:** 1 Inserm U1061, Neuropsychiatry: epidemiological and clinical research, 39 avenue Charles Flahault, BP 34493, 34093 Montpellier cedex 05, France; 2 Univ. Montpellier, U1061, Montpellier, France; 3 Department of Psychiatry, CHU Caremeau, Nîmes, France; 4 Univ. Bordeaux, Inserm, Bordeaux Population Health Research Center, UMR1219, F-33000 Bordeaux, France; 5 Department of Clinical Pharmacology, CHU Bordeaux, Bordeaux, France; 6 Centre for Clinical Brain Sciences, University of Edinburgh, Edinburgh, UK

**Keywords:** Antidepressants, Cognition, Elderly people, Cohort study

## Abstract

**Background:**

Cognitive impairment is very common in late-life depression, principally affecting executive skills and information processing speed. The aim of the study was to examine the effect of antidepressant treatment on cognitive performances over a 10-year period.

**Methods:**

The community-based cohort included 7381 participants aged 65 years and above. Five cognitive domains (verbal fluency, psychomotor speed, executive function, visuospatial skills and global cognition) were assessed up to five times over 10 years of follow-up. Treatment groups included participants under a specific antidepressant class at both baseline and the first follow-up and their follow-up cognitive data were considered until the last consecutive follow-up with a report of antidepressant use of the same class. Linear mixed models were used to compare baseline cognitive performance and cognitive decline over time according to antidepressant treatment. The models were adjusted for multiple confounders including residual depressive symptoms assessed by the Center for Epidemiologic Studies-Depression scale.

**Results:**

At baseline, 4.0% of participants were taking antidepressants. Compared to non-users, tricyclic antidepressant users had lower baseline performances in verbal fluency, visual memory and psychomotor speed, and selective serotonin reuptake inhibitor users in verbal fluency and psychomotor speed. For the two other cognitive abilities, executive function and global cognition, no significant differences were found at baseline irrespective of the antidepressant class. Regarding changes over time, no significant differences were observed in comparison with non-users whatever the cognitive domain, except for a slight additional improvement over the follow-up in verbal fluency skills for tricyclic antidepressant users.

**Conclusions:**

In this large elderly general population cohort, we found no evidence for an association between antidepressant use and post-treatment cognitive decline over 10 years of follow-up in various cognitive domains.

**Electronic supplementary material:**

The online version of this article (doi:10.1186/s12916-017-0847-z) contains supplementary material, which is available to authorized users.

## Background

Late-life depression (LLD) is a major public health issue [1]. It is associated with lower quality of life, poorer physical health and higher disability [2], as well as greater mortality risk [3]. LLD has specific characteristics, including the chronicity of symptoms [4], frequent comorbidities and the high prevalence of subsyndromal depression [5]. Cognitive impairments are very common in LLD, with executive dysfunction and decreased information processing speed being the most predominant [6–8]. Impairments in visuospatial skills and verbal fluency have also been reported as well as poor performance in episodic memory, learning and recall [8–11]. Cognitive dysfunction is furthermore predictive of poor response to pharmacological and psychological treatments of depression [12].

While there are arguments supporting antidepressant effectiveness and a favourable benefit-risk ratio for major depression [13], a much lower efficacy has been found for subsyndromal depression [14]. In particular, the potential positive effect of antidepressants on cognitive functioning in depressed elderly patients is still debated, and deleterious effects on cognition have also been reported. Several antidepressants, such as tricyclic antidepressants (TCAs), may have a detrimental impact notably due to their anticholinergic properties and selective serotonin reuptake inhibitors (SSRIs) may have a negative cognitive impact in non-responders [15]. The inappropriate use of antidepressants in subsyndromal depression may increase the risk of drug interactions and adverse reactions as aging is also associated with pharmacodynamic alterations (decreased renal clearance, altered hepatic metabolism and increased elimination half-lives). Given the risks associated with reduced cognitive competency in the elderly, the benefit-risk balance of antidepressants in relation to cognition should be given careful consideration.

In a systematic review of 43 clinical trials with a median follow-up of 8 weeks, a small effect size favouring active monotherapy was observed for verbal memory while a small effect size favouring placebo was observed for processing speed [16]. The few existing elderly community cohort studies with longer follow-up [17, 18] generally suffer from several biases notably due to limited consideration of potential confounders (e.g. use of other medications), of patterns of antidepressant use and treatment chronicity, and of channelling bias (related to underlying burden of physical and mental illness). Distinguishing the effect of depression from that of antidepressant treatment is notably a critical point [19].

The purpose of this study was to prospectively examine the association between antidepressant use and 10-year decline in five cognitive domains in a large elderly community-dwelling cohort, taking into account multiple potential confounding factors including residual depressive symptoms.

## Methods

### Study sample

Participants were recruited as part of the Three-City Cohort study of community-dwelling persons aged 65 years and over from the electoral rolls of three French cities (Bordeaux, Dijon and Montpellier) between 1999 and 2001 [20]. Of the persons contacted, the participation rate was 37%. The study protocol was approved by the Ethics Committee of the Bicêtre University-Hospital (France) and written informed consent was obtained from each participant. A standardised evaluation with a face-to-face interview and a clinical examination was undertaken at baseline and after 2, 4, 7, and 10 years.

Of the 9294 participants included in the cohort, 214 were excluded because of a diagnosis of prevalent dementia at baseline and 1159 because of they missed the 2-year follow-up examination, which was necessary for antidepressant exposure assessment as defined below. The study sample was further limited to those using the same class of antidepressant at both baseline and the 2-year visit and to those not treated with antidepressant at both visits, thus excluding a further 511 participants. Among the latter, 157 stopped taking their antidepressant between the two visits, 278 began antidepressant treatment during the 2-year period, 32 were taking two different classes of antidepressant at the same visit, and 44 changed class of antidepressant between the two visits. Twenty-nine participants missing all follow-up cognitive evaluations were also excluded. The present analyses were thus conducted on 7381 subjects. As compared with the analysed sample, the non-demented excluded participants were older (*P* < 0.0001) and more likely to have activity limitations, visual impairment and ischemic pathologies (*P* < 0.0001), respiratory diseases (*P* = 0.002), and diabetes (*P* = 0.005). They had lower levels of education and cognitive scores and higher levels of depressive and anxiety symptoms (*P* < 0.0001). They also had higher rates of use of antidepressants, benzodiazepines, anticholinergic drugs and other drugs acting on the central nervous system (*P* < 0.0001).

### Cognitive outcome measures

A battery of cognitive tests administered by a neuropsychologist assessed different cognitive domains. The Isaac’s Set Test [21] was used to provide a measure of verbal fluency or semantic access which is sensitive to changes in both frontal and temporal areas. Fluency was assessed as the total score corresponding to the sum of the number of words generated in four semantic categories within 30 seconds (animals, colours, cities, fruits). Benton’s Visual Retention Test (BVRT) [22] was used to assess visual memory, psychomotor speed and executive function were assessed using the Trail Making Tests A and B, respectively (TMTA and TMTB) (time in seconds) [23], and the Mini Mental State Examination (MMSE) was used as a global measure of cognitive function [24]. All of the cognitive tests were administered at baseline and at each wave of the follow-up, except the TMTA and B, which were not given in the 2-year wave. Consequently, and because of sparse missing data, the analyses finally involved 7364 participants for MMSE, 7262 for BVRT, 7291 for Isaac’s test, 6090 for TMTA, and 5857 for TMTB.

### Antidepressant exposure

An inventory of all drugs regularly used during the preceding month was registered at baseline and each follow-up examination. To reduce underreporting, participants were asked to provide medical prescriptions, drug packages and any other relevant material. The drugs were systematically coded using the Anatomical Therapeutic Chemical classification system. Drug exposure has previously been validated in this cohort in comparison with the reimbursement data from the healthcare insurance system. The validity of antidepressant exposure measured from participant interviews was very high, with a sensitivity of 80% and a specificity of 98% [25, 26]. Three classes of antidepressants were considered: the TCAs (non-selective monoamine reuptake inhibitors), the SSRIs and the other antidepressants, which included serotonin norepinephrine reuptake inhibitors, monoamine oxidase inhibitors and mianserin, tianeptine, viloxazine, and mirtazapine. For a given class, the treated group included participants who used this class at both baseline and the first follow-up and we considered their follow-up cognitive data until the last consecutive follow-up with a report of antidepressant use of the same class. The reference group included those who were untreated at both evaluations using their cognitive data until the last consecutive follow-up without report of antidepressant.

### Socio-demographic and clinical variables

The standardised interview included questions on socio-demographic characteristics, alcohol, caffeine, fruit and vegetable consumption, and visual impairment. Use of benzodiazepines, drugs with anticholinergic effects other than antidepressants [27], and other central nervous system drugs, as well as the total number of other medications, were derived from reported drugs (see above). Fasting blood samples were taken at baseline for glycaemia and APOE ε4 genotyping [28]. Diabetes was defined as treated or glycaemia ≥ 7 mmol/L and body mass index as weight divided by height squared. History of ischemic pathologies (stroke, angina pectoris, myocardial infarction and cardiovascular surgery) was established according to standardised questions. Respiratory pathologies included self-reported chronic bronchitis and asthma attacks (over the last 12 months). Potential cases of dementia were reviewed by an independent committee of neurologists in order to obtain a consensus on the diagnosis according to the DSM-IV criteria. A hierarchical indicator of disability [29] combined three scales, namely the Rosow and Breslau mobility scale [30], Lawton-Brody Instrumental Activity of Daily Living (IADL) scale [31], and Katz’s Activity of Daily Living (ADL) scale [32]. This indicator defines four levels of disability: full independence, mild disability (only mobility restriction), moderate disability (mobility and IADL limitation), and severe disability (mobility, IADL and ADL limitation). Severity of depressive symptoms was assessed by the Center for Epidemiologic Studies-Depression scale (CES-D) [33] and history of major depressive episode (MDE) was diagnosed using the Mini International Neuropsychiatric Interview [34]. Spielberger’s State-Trait Anxiety Inventory was used to measure trait anxiety symptoms [35].

### Statistical analyses

Comparison of baseline characteristics between included and excluded participants as well as between antidepressant users and non-users was performed using χ^2^ tests and unpaired Wilcoxon rank-sum tests. We used linear random-effect models to analyse the association between antidepressant use and 10-year change on cognitive scores taken as continuous variables. In order to normalise the distributions, cognitive variables were transformed using (15-Benton)^1/2^, (30-MMSE)^1/2^ and natural logarithm of TMT. Each model included time, antidepressant group, time*antidepressant group interaction and covariates. In the tables, the term ‘antidepressant’ represents the intercept point differences on cognitive scores and corresponds to the baseline differences between antidepressant treated and untreated groups. The term ‘time’ indicates the linear evolution per year on the cognitive test. The term for interaction ‘antidepressant*time’ represents the additional annual modification on the selected cognitive tests for antidepressant users expressed as a score change per year.

We used multiple imputations to permit multivariate analysis of all participants who had baseline and at least one follow-up cognitive evaluation. We generated five replications of the original data set, in which missing values (2.1% of data) for 17 covariates considered in the analysis were replaced by values generated according to the Markov Chain Monte Carlo method [36] using the PROC MI SAS procedure. Each imputed data set was then analysed using the linear mixed models described above and the results were pooled to calculate mean estimates and their standard error using the PROC MIANALYZE procedure.

To control for confounding effects, two nested models were examined. The first model was adjusted for sex, centre, baseline age, and education and for significant interactions: age*time, centre*time, and education*time. The multi-adjusted model included additional covariates and their time interactions (with age, centre, education and APOE ε4) that were associated with at least one of the cognitive scores (*P* ≤ 0.10 in the first model). We also conducted three sensitivity analyses, namely (1) excluding paroxetine users from the SSRI group as this drug has been reported to have a high anticholinergic activity in comparison with other SSRIs [37], (2) excluding participants with history of MDE to reduce the impact of past depression on cognition, and (3) excluding participants with incident dementia to control for the protopathic bias where antidepressants are prescribed for an early manifestation of a dementia not yet diagnostically detected. A last analysis was performed keeping in the models the follow-up cognitive data after antidepressant treatment changes in order to have similar lengths of follow-up between groups. All analyses were conducted using the statistical software SAS version 9.4 for Windows.

## Results

### Subject characteristics

Within this elderly community-dwelling sample, 296 of the 7381 participants (4.0%) were taking the same class of antidepressants at both baseline and the 2-year visit, of whom 89 (1.2%) used TCAs, 159 (2.2%) SSRIs, and 48 (0.7%) other antidepressants. Nearly all baseline characteristics were associated with antidepressant use, except for body mass index, respiratory diseases, ischemic pathologies, and ApoE ε4. At baseline, median CES-D and anxiety scores were significantly higher in those prescribed antidepressants while all cognitive performances were lower except MMSE (Table 1).

**Table 1:** Characteristics of the study population according to antidepressant medication use, *n* = 7381

	Antidepressant use at baseline and 2-year follow-up		
	Non treated	Treated within the same class	χ^2^
	*N* = 7085 %	*N* = 296 %	*P* value
Gender (women)	59.4	79.7	<0.0001
Education			
< 6 years	23.9	28.4	0.05
6–11 years	36.0	38.2	
> 11 years	40.1	33.4	
Body mass index			
Normal (<25)	47.4	43.6	0.28
Overweight (25–30)	39.8	40.9	
Obese (≥30)	12.8	15.5	
Alcohol			
0	19.1	29.7	<0.0001
1–36 g/day	72.0	67.2	
> 36 g/day	8.9	3.1	
Caffeine			
0–1 cup/day	25.2	34.8	0.001
2–3 cups/day	59.8	52.0	
> 3 cups/day	15.0	13.2	
Fruit and vegetable consumption			0.0006
Less than twice per day	28.3	37.5	
Hierarchical disability indicator			<0.0001
Fully independent	55.8	31.8	
Mild disability	37.9	49.0	
Moderate to severe disability	6.3	19.2	
Visual impairment	15.8	24.7	<0.0001
Respiratory pathologies	8.0	7.4	0.73
Diabetes	9.6	13.8	0.01
Ischemic pathologies	15.8	20.6	0.06
ApoE 4 allele	20.2	19.9	0.92
Benzodiazepine use	17.5	59.8	<0.0001
Anticholinergic drug use	5.3	13.2	<0.0001
Other CNS drugs	5.3	18.2	<0.0001
Number of other medications			<0.0001
0–1	34.9	18.9	
2	29.5	33.5	
3+	35.6	47.6	
	Median (IQR)	Median (IQR)	Wilcoxon test *P* value
Age	73 (69–77)	74 (70–78)	0.007
CES-D score	8 (3–14)	16 (7–24)	<0.0001
Spielberger score	38 (33–45)	47 (40–54)	<0.0001
MMSE score	28 (27–29)	28 (26–29)	0.09
Benton test score	12 (10–13)	11 (9–13)	<0.0001
Isaacs total score	48 (41–55)	43 (37–50)	<0.0001
TMTA (seconds)	50 (40–64)	60 (48–75)	<0.0001
TMTB (seconds)	97 (75–129)	118 (85–160)	<0.0001

*CES-D* Center for Epidemiologic Studies-Depression scale, *CNS* central nervous system, *IQR* inter-quartile range, *MMSE* Mini Mental State Examination, *TMTA* and *TMTB* Trail Making Tests A and B

### Antidepressant use and 10-year cognitive decline

The median (IQR) follow-up time was 8.0 (3.7–9.0) years for participants not treated and 3.7 (1.9–7.2) for those treated with antidepressants at both baseline and the 2-year follow-up (*P* = 0.0001). Of the selected sample, 3471 (47.0%) participants had cognitive assessments at each of the five waves. The results of the minimally adjusted models are given in Table 2. Regarding the term ‘antidepressant’ (baseline differences), TCA and SSRI users had lower performances than the untreated group on BVRT, Isaac’s test and TMTA. An association was also found at baseline between SSRIs and TMTB. Other antidepressant use was also associated with lower baseline performances on the Isaac’s test and TMTA. Regarding the slope with time (interaction term), only the TCA group was associated with a slight improvement on the Isaac’s test compared with non-users.

**Table 2:** Minimally adjusted associations of antidepressant use with 10-year cognitive changes

	MMSE $\sqrt{30-\mathrm{MMSE}}$ *N* = 7364		BVRT $\sqrt{15-\mathrm{BVRT}}$ *N* = 7262		Isaac’s test *N* = 7291		TMTA *ln*(*TMTA*) *N* = 6090		TMTB *ln*(*TMTB*) *N* = 5857	
	β (SE)	*P* value	β (SE)	*P* value	β (SE)	*P* value	β (SE)	*P* value	β (SE)	*P* value
Antidepressant		0.14		<0.0001		<0.0001		<0.0001		0.004
TCAs	0.110 (0.064)	0.08	0.224 (0.054)	<0.0001	–5.96 (0.97)	<0.0001	0.170 (0.044)	0.0001	0.094 (0.051)	0.07
SSRIs	0.069 (0.048)	0.15	0.128 (0.041)	0.002	–3.41 (0.74)	<0.0001	0.131 (0.034)	0.0001	0.117 (0.039)	0.003
Others	–0.052 (0.086)	0.55	0.095 (0.073)	0.19	–2.61 (1.34)	0.05	0.141 (0.061)	0.02	0.082 (0.071)	0.25
Antidepressant*time		0.53		0.56		0.20		0.39		0.33
TCAs*time	0.012 (0.014)	0.38	0.006 (0.012)	0.65	0.31 (0.15)	0.04	–0.011 (0.007)	0.11	–0.006 (0.009)	0.48
SSRIs*time	0.010 (0.011)	0.36	0.013 (0.009)	0.17	0.08 (0.12)	0.50	–0.002 (0.005)	0.75	–0.001 (0.006)	0.83
Others*time	0.016 (0.020)	0.43	0.002 (0.018)	0.89	–0.06 (0.22)	0.77	0.006 (0.010)	0.53	–0.022 (0.013)	0.09

Models adjusted for age, sex, centre, education and time by age, time by centre and time by education interactions and with non-users as the reference group.

*BVRT* Benton’s Visual Retention Test, *MMSE* Mini Mental State Examination, *SE* standard error, *SSRI* selective serotonin reuptake inhibitors, *TCA* tricyclic antidepressant, *TMTA* and *TMTB* Trail Making Tests A and B

When potential confounders were further included in the linear random-effect models (Table 3), the TCA group was still associated at baseline with lower performances on the Isaac’s test (*P* < 0.0001), BVRT (*P* = 0.005) and the TMTA (*P* = 0.04), compared to non-users. This corresponds to approximately 4.2 words less on the Isaac’s score, 0.6 point less on the BVRT score (for a median BVRT of 12) and 4.7 seconds more on the TMTA time (for a median TMTA of 50) (see footnote in Table 3 for back transformations in original scale). The SSRI users showed, at baseline, a mean of 1.6 words less on the Isaac’s test (*P* = 0.03) and an increased time on TMTA (*P* = 0.05), which corresponds to 3.4 seconds (for a median time of 50), whereas the associations with baseline BVRT and TMTB were no longer significant. The associations for other antidepressants were also no longer significant.

**Table 3:** Multi-adjusted associations of antidepressant use with 10-year cognitive changes

	MMSE $\sqrt{30-\mathrm{MMSE}}$ *N* = 7364		BVRT $\sqrt{15-\mathrm{BVRT}}$ *N* = 7262		Isaac’s test *N* = 7291		TMTA *ln*(*TMTA*) *N* = 6090		TMTB *ln*(*TMTB*) *N* = 5857	
	β (SE)	*P* value	β (SE)	*P* value	β (SE)	*P* value	β (SE)	*P* value	β (SE)	*P* value
Antidepressant		0.46		0.02		<0.0001		0.04		0.55
TCAs	0.034 (0.064)	0.59	0.153 (0.054)	0.005	–4.19 (0.96)	<0.0001	0.090 (0.043)	0.04	0.018 (0.051)	0.71
SSRIs	–0.018 (0.048)	0.70	0.053 (0.041)	0.19	–1.58 (0.74)	0.03	0.066 (0.034)	0.05	0.056 (0.039)	0.16
Others	–0.126 (0.086)	0.14	0.029 (0.073)	0.69	–0.78 (1.33)	0.56	0.050 (0.060)	0.40	0.005 (0.070)	0.94
Antidepressant*time		0.52		0.50		0.18		0.38		0.32
TCAs*time	0.012 (0.014)	0.40	0.005 (0.012)	0.69	0.32 (0.15)	0.04	–0.011 (0.007)	0.11	–0.006 (0.009)	0.51
SSRIs*time	0.011 (0.011)	0.31	0.014 (0.009)	0.14	0.08 (0.12)	0.51	–0.002 (0.005)	0.77	–0.001 (0.006)	0.86
Others*time	0.015 (0.020)	0.45	0.002 (0.018)	0.91	–0.06 (0.22)	0.78	0.006 (0.010)	0.52	–0.023 (0.013)	0.08

Models adjusted for time, age, sex, centre, education, body mass index, alcohol, caffeine, fruit and vegetable consumption, activity limitations, visual deficiency, respiratory disease, diabetes, ischemic pathology, ApoE4 genotype, benzodiazepines, anticholinergic drugs, other psychotropic drugs, number of other drugs, depressive symptoms, anxiety symptoms as well as time by age, time by centre, time by education and time by ApoE4 interactions. Non-users as the reference group

To transform back to the original scale the difference (Δ = *y*_2_ − *y*_1_) between a group and the non-users:

MMSE: $\Delta =-{\beta}^2-2\beta \sqrt{\left(30-{y}_1\right)}$; BVRT: $\Delta =-{\beta}^2-2\beta \sqrt{\left(15-{y}_1\right)}$; TMTA et TMTB: Δ = *exp*(*β*) *y*_1_ − *y*_1_

*BVRT* Benton’s Visual Retention Test, *MMSE* Mini Mental State Examination, *SE* standard error, *SSRI* selective serotonin reuptake inhibitors, *TCA* tricyclic antidepressant, *TMTA* and *TMTB* Trail Making Tests A and B

Regarding antidepressant by time interaction, the overall *P* values were all above 0.05; only the TCA group was associated with a slight improvement over time for the Isaac’s test (*P* = 0.04) of 0.3 words by year. No significant interactions between antidepressant use and sex were observed for all of the cognitive tests (*P* > 0.20).

### Sensitivity analyses

When the users of paroxetine were excluded (*n* = 66) from the SSRI group, the associations at baseline with the Isaac’s test (*P* = 0.09) and the TMTA (*P* = 0.93) became non-significant (data not shown). Excluding the participants with a past history of MDE strengthened the results for Isaac’s test and TMTA at baseline (Additional file 1: Table S1), the association between SSRIs and baseline TMTB was again significant (*P* = 0.02), whereas that of TCAs with baseline BVRT became non-significant (*P* = 0.18). Regarding the declines over time, the overall tests were still not significant (*P* > 0.26). When the participants with incident dementia were excluded (*n* = 685) the TCA group was still associated with lower performances at baseline on the Isaac’s test (*P* < 0.0001) and an improved score over time (*P* = 0.01) but the other associations were no longer significant (Additional file 1: Table S2).

Finally, as censoring after treatment discontinuation generated differences in follow-up time between treated and not treated groups, additional analyses were performed without censoring (Additional file 1: Table S3). The median (IQR) follow-up time was 8.4 (4.0–9.0) and 7.6 (3.7–9.1) years for participants not treated and treated, respectively (*P* = 0.08). The same results were observed except that the improvement over time in the TCA group for the Isaac’s test was not significant (*P* = 0.31).

## Discussion

In this large prospective study, 4.0% of community-dwelling elderly people were taking antidepressants, mainly SSRIs and TCAs. Differences in baseline performance levels and decline over time were observed according to cognitive domains with SSRIs and TCAs but not with the other antidepressants. Compared to non-users, the TCA users showed lower baseline performances of 9% for verbal fluency, 5% for visual memory (BVRT) and 9% for psychomotor speed (TMTA), and the SSRI users of 3% for verbal fluency and 7% for psychomotor speed. On the other hand, no significant differences were found at baseline for global cognitive performances (MMSE) or executive function (TMTB) irrespective of the treatment groups. Regarding changes over time, only a slow additional improvement (0.7% per year) was observed on verbal fluency for the TCA group, which was not significant when the analyses were performed without censoring cognitive data. Hence, regardless of the cognitive domains, we did not observe statistically significant accelerated cognitive decline over time in treated participants, meaning that the differences found at inclusion remained constant over the 10-year follow-up.

Hence, our study indicates that TCAs and SSRIs are principally associated with relatively weak cognitive impairment at baseline, mainly related to verbal fluency, visual memory and psychomotor skills, but the question remains as to whether this occurred before or at treatment initiation. Impairment in executive function, psychomotor speed and, although less consistently, verbal fluency have been associated with depression [6–8, 11]. Our results remained significant after adjustment for other possible co-determinants of cognitive impairment, including current anxiety and depressive symptoms, and also when participants with past history of MDE were excluded, suggesting that the antidepressants themselves rather than the underlying burden of illness could be associated with impaired cognitive performance. However, the effect of TCAs on baseline cognitive visual memory may be more related to psychological comorbidity as it became statistically non-significant when participants with a history of MDE were excluded. Baseline cognitive impairment may be the consequence of the unknown level of depressive symptoms just before initiation of treatment and of a limited capacity of antidepressants to improve cognitive performance. It is thus difficult to disentangle the initial effect of treatment on cognitive performances from that of depression prior to inclusion. Impairment in executive function is also a common hallmark of poor responders to psychopharmacologic interventions, which may persist even after treatment cessation [7, 38]. We observed lower executive function only in participants treated with SSRIs and without history of MDE. Whether this may correspond to under-treated or resistant late-onset depression remains to be determined. Antidepressants, and in particular SSRIs, may also be prescribed for symptoms associated with suspected mild cognitive impairment. In this prodromal dementia group, the cognitive deficit may persist over time. In line with this hypothesis, when we removed the participants with incident dementia, the baseline associations were weakened and remained only significant for TCAs and the Isaac’s test.

In our study, TCA and SSRI intake were not associated with substantial accelerated cognitive decline over time after multiple adjustments including residual depressive symptoms. So far, few large prospective studies have examined the relationship between antidepressant use and cognitive decline in elderly populations. These studies had, however, probable confounding bias (see above) and they mainly focused on global cognitive change on the MMSE, which is highly likely to have a ceiling effect in non-demented participants. Our results support and extend to specific cognitive domains and longer follow-up, recent data of a nationally representative cohort of US residents showing that antidepressant use did not modify the course of 6-year global cognitive change [18]. In a poorly adjusted analysis of a Canadian prescription database, antidepressant use was also not associated with global cognitive changes from baseline among patients with or without major depression, but was moderately associated with an MMSE score increase over time in patients with minor depression [39]. Conversely, both SSRIs and TCAs were associated with an increased risk of mild cognitive impairment in a large cohort of elderly women [17]. However, this study did not take into account possible confounding by other psychotropic drugs and potential prodromal dementia symptoms. In our study, the main confounders for baseline differences were depressive symptom score, anxiety score and benzodiazepine use. Additionally, people taking drugs with anticholinergic properties are known to be at increased risk for cognitive decline and dementia [27], and this has rarely been considered previously. Antidepressants, mainly TCAs, but also the SSRI paroxetine, may have high anticholinergic effects [37]. In our study, when paroxetine was removed from the SSRI group, the associations with baseline verbal fluency and psychomotor speed became non-significant, suggesting that paroxetine may be the principle compound responsible for the effect of SSRIs at baseline. However, the lower statistical power in the restricted group precludes drawing a definite conclusion.

The study has several strengths. First, the multicentric longitudinal design and the size of the sample with more than 7300 elderly participants from the general population, of whom half were followed for 10 years. Second, antidepressant use was ascertained at baseline and during follow-up by examining the prescriptions and boxes, thus minimising exposure misclassification. Exposure to both current and chronic antidepressant medication has previously been shown within this cohort to be highly valid in comparison with the reimbursement data from the healthcare insurance system [25, 26]. Third, although in observational studies residual confounding may always subsist, our analyses overcame several limitations of previous studies. To our knowledge, this is the first study which adjusted for such a large range of different key factors, including ApoE4 genotype, behavioural characteristics, activity limitations, physical and mental health as well as anticholinergic drugs and other psychotropic drugs. Fourth, the linear mixed models took into account the influence of confounders on cognitive changes including all significant terms for interactions with time (age, education, ApoE4). Sixth, the cognitive evaluation included a battery of tests examining different and complementary cognitive domains.

Our study has several limitations. No information about dose and frequency of reported treatment was available in our study nor on the duration and type of treatment before inclusion. The antidepressant treatment was initiated before inclusion of participants in the present study and the cognitive state was unknown at treatment initiation. We thus cannot conclude if the observed baseline loss of performance is due to an effect that rapidly appears after treatment onset without further worsening over the follow-up or to the consequence of pre-treatment depression, which we could not fully control. Studying each cognitive test separately conducted to statistical multiple testing and we did not correct for inflated Type 1 error rates. However, each selected cognitive test examined different cognitive domains. Using linear mixed models, we assumed that dropouts and missing data were missing at random [40]; however, some dropouts may be related to unexpected cognitive decline and this can lead to under- or overestimated effects. Finally, bias could have been introduced by the exclusion of participants with incomplete information on exposure who were more likely to be frail and thus susceptible to cognitive decline.

## Conclusions

In this large elderly general population cohort, we found that antidepressant use was not significantly associated with cognitive decline in various domains of cognitive abilities over 10 years of follow-up. However, treated participants presented baseline deficits which remained constant over time mainly in verbal fluency and psychomotor functions known to be affected by depression. These findings could be compatible with an early but still clinically significant decline of cognitive functioning at antidepressant initiation, which, however, would not further progress over a 10-year period. While only a minority of patients with LLD yet receive a treatment for depression [41], our findings did not provide evidence for an association between antidepressant use and post-treatment cognitive decline in elderly people.

## Additional file

Additional file 1: Table S1. Multi-adjusted associations of baseline antidepressant use with 10-year cognitive changes in participants without past major depressive episode. **Table S2.** Multi-adjusted associations of baseline antidepressant use with 10-year cognitive changes in participants without incident dementia. **Table S3.** Multi-adjusted associations of antidepressant use with 10-year cognitive changes without censoring of cognition after treatment change. (DOCX 26 kb)

## Abbreviations

ADL: activity of daily living
BVRT: Benton’s Visual Retention Test
CES-D: Center for Epidemiologic Studies-Depression scale
IADL: instrumental activity of daily living
IQR: interquartile range
LLD: late-life depression
MDE: major depressive episode
MMSE: Mini Mental State Examination
SSRI: selective serotonin reuptake inhibitors
TCA: tricyclic antidepressant
TMTA and TMTB: Trail Making Tests A and B
