# Supplementary material for: Antidepressant use and cognitive decline in community-dwelling elderly people – The Three-City Cohort
Source: BMC Med. 2017 Apr 19;15:81. doi: 10.1186/s12916-017-0847-z (PMC5397783; doi:10.1186/s12916-017-0847-z)
Supplement: Additional file 1: Table S1. — Multi-adjusted associations of baseline antidepressant use with 10-year cognitive changes in participants without past major depressive episode. Table S2. Multi-adjusted associations of baseline antidepressant use with 10-year cognitive changes in participants without incident dementia. Table S3. Multi-adjusted associations of antidepressant use with 10-year cognitive changes without censoring of cognition after treatment change. (DOCX 26 kb) [file 12916_2017_847_MOESM1_ESM.pdf]

Table S1. Multi-adjusted associations of antidepressant use with 10-year cognitive changes in participants without past major depressive episode

	MMSE $\sqrt{30 - MMSE}$ N=6036		BVRT $\sqrt{15 - BVRT}$ N=5964		Isaac's test N=5981		TMTA $ln(TMTA)$ N=5099		TMTB $ln(TMTB)$ N=4928	
	β (SE)	p-value	β (SE)	p-value	β (SE)	p-value	β (SE)	p-value	β (SE)	p-value
**Antidepressant**		0.95		0.30		0.0007		0.001		0.10
**TCAs**	0.006 (0.090)	0.95	0.104 (0.077)	0.18	-4.545 (1.370)	0.0009	0.191 (0.063)	0.002	0.050 (0.075)	0.51
**SSRIs**	-0.005 (0.058)	0.93	0.061 (0.050)	0.22	-1.971 (0.899)	0.03	0.102 (0.040)	0.01	0.115 (0.048)	0.02
**Others**	-0.065 (0.111)	0.56	-0.054 (0.095)	0.57	-2.472 (1.735)	0.15	0.055 (0.078)	0.48	-0.016 (0.093)	0.87
**Antidepressant*time**		0.66		0.72		0.41		0.56		0.26
**TCAs*time**	0.019 (0.022)	0.40	0.007 (0.020)	0.71	0.395 (0.236)	0.09	-0.016 (0.011)	0.18	-0.006 (0.015)	0.67
**SSRIs*time**	0.012 (0.013)	0.35	0.012 (0.012)	0.29	-0.012 (0.140)	0.93	0.001 (0.006)	0.92	-0.015 (0.008)	0.06
**Others*time**	0.002 (0.025)	0.94	0.006 (0.022)	0.78	-0.063 (0.275)	0.82	0.005 (0.013)	0.69	-0.008 (0.016)	0.61

Models adjusted for time, age, gender, center, education, body mass index, alcohol, caffeine, fruit and vegetable consumption, activity limitations, visual deficiency, respiratory disease, diabetes, ischemic pathology, ApoE4 genotype, benzodiazepines, anticholinergic drugs, other psychotropic drugs, number of other drugs, depressive symptoms, anxiety symptoms as well as time by age, time by center, time by education and time by ApoE4 interactions.

BVRT: Benton's Visual Retention Test, MMSE: Mini Mental State Examination, SE: standard error, SSRI: selective serotonin reuptake inhibitors, TCA: tricyclic antidepressant, TMTA and TMTB: Trail Making Tests A and B

Table S2. Multi-adjusted associations of antidepressant use with 10-year cognitive changes in participants without incident dementia

	MMSE $\sqrt{30 - MMSE}$ N=6681		BVRT $\sqrt{15 - BVRT}$ N=6594		Isaac's test N=6622		TMTA $ln(TMTA)$ N=5577		TMTB $ln(TMTB)$ N=5408	
	β (SE)	p-value	β (SE)	p-value	β (SE)	p-value	β (SE)	p-value	β (SE)	p-value
**Antidepressant**		0.59		0.23		0.0007		0.08		0.66
**TCAs**	-0.019 (0.071)	0.79	0.114 (0.061)	0.06	-4.272 (1.073)	<0.0001	0.085(0.048)	0.08	0.006 (0.056)	0.91
**SSRIs**	-0.057 (0.052)	0.27	0.035 (0.044)	0.44	-0.776 (0.793)	0.33	0.056 (0.036)	0.12	0.051 (0.043)	0.23
**Others**	-0.078 (0.092)	0.40	0.040 (0.078)	0.61	-0.975 (1.413)	0.49	0.077 (0.063)	0.22	-0.025 (0.075)	0.74
**Antidepressant*time**		0.78		0.63		0.02		0.60		0.76
**TCAs*time**	0.011 (0.015)	0.48	0.007 (0.014)	0.59	0.420 (0.162)	0.01	-0.010 (0.008)	0.20	-0.005 (0.010)	0.63
**SSRIs*time**	0.008(0.011)	0.46	0.012 (0.010)	0.24	0.209 (0.121)	0.09	-0.003 (0.005)	0.64	0.003 (0.007)	0.64
**Others*time**	0.005 (0.020)	0.82	0.006 (0.019)	0.76	-0.111 (0.221)	0.62	-0.0003 (0.010)	0.98	-0.012(0.014)	0.40

Models adjusted for time, age, gender, center, education, body mass index, alcohol, caffeine, fruit and vegetable consumption, activity limitations, visual deficiency, respiratory disease, diabetes, ischemic pathology, ApoE4 genotype, benzodiazepines, anticholinergic drugs, other psychotropic drugs, number of other drugs, depressive symptoms, anxiety symptoms as well as time by age, time by center, time by education and time by ApoE4 interactions.

BVRT: Benton's Visual Retention Test, MMSE: Mini Mental State Examination, SE: standard error, SSRI: selective serotonin reuptake inhibitors, TCA: tricyclic antidepressant, TMTA and TMTB: Trail Making Tests A and B

Table S3. Multi-adjusted associations of antidepressant use with 10-year cognitive changes without censoring of cognition after treatment change

	MMSE<br>$\sqrt{30 - MMSE}$<br>N=7365		BVRT<br>$\sqrt{15 - BVRT}$<br>N=7263		Isaac's test<br>N=7293		TMTA<br>$ln(TMTA)$<br>N=6380		TMTB<br>$ln(TMTB)$<br>N=6128	
	β (SE)	p-value	β (SE)	p-value	β (SE)	p-value	β (SE)	p-value	β (SE)	p-value
**Antidepressant**		0.34		0.01		0.0002		0.02		0.54
**TCAs**	0.035 (0.063)	0.58	0.156 (0.053)	0.003	-3.98 (0.96)	<0.0001	0.080 (0.037)	0.03	0.022(0.043)	0.61
**SSRIs**	-0.027 (0.047)	0.57	0.062 (0.040)	0.12	-1.33 (0.73)	0.07	0.067 (0.029)	0.03	0.047 (0.034)	0.17
**Others**	-0.139 (0.084)	0.10	0.033 (0.071)	0.65	-0.77 (1.32)	0.56	0.016 (0.051)	0.75	-0.008 (0.060)	0.90
**Antidepressant*time**		0.19		0.31		0.51		0.58		0.75
**TCAs*time**	0.009 (0.011)	0.39	0.0003 (0.010)	0.98	0.12 (0.12)	0.31	-0.005 (0.005)	0.35	0.003 (0.007)	0.61
**SSRIs*time**	0.014 (0.008)	0.10	0.014 (0.007)	0.06	-0.09 (0.09)	0.32	0.002 (0.004)	0.65	0.001 (0.005)	0.86
**Others*time**	0.018 (0.015)	0.23	0.003 (0.013)	0.78	-0.09 (0.17)	0.60	0.007 (0.007)	0.35	-0.008 (0.009)	0.35
